# Criteria for assessing climate change impacts on ecosystems

**DOI:** 10.1002/ece3.7

**Published:** 2011-09

**Authors:** Craig Loehle

**Affiliations:** National Council for Air and Stream Improvement Inc.Washington Street, Naperville, Illinois, USA

**Keywords:** Biodiversity, climate envelope model, extinction risk, General Circulation Model, impact assessment

## Abstract

There is concern about the potential impacts of climate change on species and ecosystems. To address this concern, a large body of literature has developed in which these impacts are assessed. In this study, criteria for conducting reliable and useful assessments of impacts of future climate are suggested. The major decisions involve: clearly defining an emissions scenario; selecting a climate model; evaluating climate model skill and bias; quantifying General Circulation Model (GCM) between-model variability; selecting an ecosystem model and assessing uncertainty; properly considering transient versus equilibrium responses; including effects of CO_2_ on plant response; evaluating implications of simplifying assumptions; and considering animal linkage with vegetation. A sample of the literature was surveyed in light of these criteria. Many of the studies used climate simulations that were >10 years old and not representative of best current models. Future effects of elevated CO_2_ on plant drought resistance and productivity were generally included in growth model studies but not in niche (habitat suitability) studies, causing the latter to forecast greater future adverse impacts. Overly simplified spatial representation was frequent and caused the existence of refugia to be underestimated. Few studies compared multiple climate simulations and ecosystem models (including parametric uncertainty), leading to a false impression of precision and potentially arbitrary results due to high between-model variance. No study assessed climate model retrodictive skill or bias. Overall, most current studies fail to meet all of the proposed criteria. Suggestions for improving assessments are provided.

## Introduction

The potential future impacts of climate change are of increasing concern. Impacts on ecosystems could potentially affect the capacity of natural systems to produce wood products, crops, livestock, and game. There is also concern about impacts on biodiversity in general and species extinctions in particular.

To assess these risks, a voluminous literature has appeared in which ecosystem or species responses to future possible climates are evaluated. These studies often utilize the experimental literature as a basis for longer term extrapolation. Two basic approaches have been used to evaluate long-term natural system response (whereas crops can be evaluated experimentally and are not considered further here): habitat suitability (niche) models and simulations. In habitat suitability models, the climate space (with or without consideration of soils, topography, etc.) where a species or vegetation type is currently found is characterized by some sort of statistical model, and this model is applied to a future climate scenario (e.g., Aitken et al. 2008). In simulations, a tree, stand, population, or ecosystem growth model is applied to current and scenario cases and the results are compared (e.g., Coops and Waring 2011).

While the literature using these approaches is vast, it is not systematic or standardized. Many choices must be made during the analysis that can lead to large and arbitrary differences in study outcomes. For example, Cramer et al. (2001), using six dynamic global vegetation models, gave a range of outcomes for the global carbon sink in 2100 under rising CO_2_ and climate change of 0.3 to 6.6 Pg C y^–1^, an immense range of 22×. Verburg et al. (2011) showed that spatial data errors in mapped attributes can produce larger uncertainties than the process under investigation. Further examples are cited in later sections herein. Furthermore, it will be shown herein that certain key factors have been left out of many analyses, potentially affecting their interpretation. Such issues can lead to the observed widespread failures of model forecasting (e.g., Pilkey–Jarvis and Pilkey 2008).

The purpose of this study, then, is to propose criteria for developing ecosystem assessments of response to climate change. These criteria involve the choices that must be made at each step of the assessment process. In some cases, it is sufficient to make explicit the limits that a particular choice (e.g., emissions scenario) places on what can be inferred from the study. In others, including or excluding a particular factor or model can create a particular bias that must be taken into account. For example, if a particular climate model predicts too much or too little rainfall in the 20th century base runs compared to actual rainfall, this will bias or distort results of an ecosystem simulation. The presentation of criteria is followed by an assessment of the extent to which the criteria have been addressed in recent literature.

## Ecosystem Assessment Criteria

The assessment of any ecosystem impact involves a series of steps, especially when long-term effects are being evaluated. Failure to exercise care in this process can lead to underestimation of uncertainty and even to arbitrary results. In order to avoid such negative outcomes, a minimal set of criteria is proposed for some critical steps in this process. More detailed criteria could be developed (e.g., for statistical testing), but this minimal set is sufficient for the present analysis. The criteria for decisions that must be made to put together a complete and valid assessment involve the following:

1. Clearly define emissions scenario.
2. Select climate model(s).
3. Evaluate climate model skill and bias.
4. Quantify General Circulation Model (GCM) between-model variability.
5. Select an ecosystem model and assess uncertainty.
6. Properly consider transient versus equilibrium responses.
7. Include effects of CO_2_ on plant response.
8. Evaluate implications of simplifying assumptions.
9. Consider animal linkage with vegetation.

Each of these criteria is discussed more fully in what follows.

### Clearly defining an emissions scenario

It is standard practice to evaluate ecosystem impacts in terms of the Intergovernmental Panel on climate change standard scenarios for future emissions of greenhouse gases (Intergovernmental Panel on Climate Change [IPCC] 2007). The first factor to consider is that both the scenarios themselves and the model ensemble warming due to the scenarios differ between the IPCC 2001 (Intergovernmental Panel on Climate Change [IPCC] 2001) and 2007 reports. For example, while the A2 2001 scenario is projected to produce 3.8°C warming by 2100, the 2007 A2 scenario only projects 3.4°C global mean increase by 2100. Thus, studies using the “same” scenario published at earlier or later dates will not be strictly comparable. In addition, model runs from 2001 show wider spread than those from the 2007 report. In particular, earlier studies are likely to show more impact and should be considered to be superseded by more recent ones. Model outputs from earlier scenarios and GCMs may still be available but should no longer be used and will exaggerate impacts if used.

A second criterion for utilizing scenarios is to distinguish between worst-case scenario and most likely cases. If the worst-case scenario is used in the assessment, it should not be stated that the simulated impact is “what will” happen, but rather is a worst case. If evaluation of a likely scenario is desired, the appropriate case should be used.

### Selecting a climate model

Early (before 1995 or even up to 2000+) GCMs either did not simulate precipitation or were known to do so poorly (Corte–Real et al. 1995). This includes most of the models used in the 2001 IPCC report. For evaluating ecosystem impacts, this limitation cannot be addressed by using a constant precipitation regime. If precipitation is held constant and temperature is increased, one obviously would predict negative effects on plants. Theory predicts, in contrast, that a warmer climate will be accompanied by increased precipitation (Wentz et al. 2007; Schliep et al. 2010; Stephens et al. 2010). Whether increased evaporative demand will be balanced by increased precipitation is an open question and one on which different models do not necessarily agree. The future predicted water balance will also vary regionally, from wetter to drier to neutral. Thus, it is critical that older GCM models/model runs no longer be used for any ecosystem studies that involve water balance. Further, in literature summaries, older studies should not be given equal weight to those using more recent GCM results.

### Evaluating climate model skill and bias

Output from a climate model is often used to evaluate ecosystem response, perhaps with a regional downscaling first. These outputs are virtually always taken at face value for conducting impact studies. Any particular model, however, may have known skill and bias issues with respect to regional climates. Skill can be evaluated by matching the pattern in time or distribution of temperatures or rainfall between the model and historical data. For example, a GCM might produce skillful annual precipitation amounts, but the seasonal distribution could be very different from actual. The seasonal amounts could be critical to properly simulating plant growth. Similarly, a GCM could consistently predict temperatures too warm for a region, even though matching historical trends. This could lead to modeled impacts that are not realistic. In IPCC assessments, in which global trends over 100 years are being evaluated, these skill and bias issues may not affect the calculation of trends, but at the regional/local scale for simulating ecosystem response, they cannot be ignored.

Anagnostopoulos et al. (2010) compared six climate models at multiple scales to weather data for the contiguous United States. Model results were all warmer than the observed mean annual temperature and minimum monthly temperature by up to 4°C. This bias creates an obvious problem for any bioclimatic (niche) or simulation model used to forecast future distributions because absolute temperatures, not just trends, are critical to biological processes. This is particularly true when models are developed using actual (not model) climate data, which do not share the model bias. Likewise, at the continental scale, they found modeled precipitation to be 36% above the true value, which has implications for any biological model. Schliep et al. (2010) and Stephens et al. (2010) noted multiple failings in the ability of GCMs to model precipitation, including both bias and lack of skill.

Consider a climate model that produces output at the dry end of what occurs in a particular region. A model of forest growth is run under constant climate, but because of the climate model's temperature bias, the simulated forest is near the point at which drought stress would cause dieback. Under almost any additional warming, this simulated forest using this climate model would show dieback. This bias can be addressed by comparing the GCM output to local weather data and adjusting the model bias (as Ines and Hansen 2006) before simulating forest growth (for both control and impact scenarios). If it is determined that skill is lacking (e.g., rainfall frequency distribution is wildly wrong or seasonal temperatures are not proportional) then another model should be used. In any case, climate model adequacy for the intended purpose should be evaluated rather than treating model output as if it were “data.”

### Quantifying GCM between-model variability

GCMs exhibit considerable variation in outputs (Furrer et al. 2007), especially at regional scales (e.g., Rowell 2006). For example, Woollings (2010) found that simulations for Europe differed between models (and from actual weather) for the jet stream location, zonal air flow, blocking highs, and other key weather patterns. It is important to note that models do not differ only in trends or stochastically, but also in terms of seasonality and spatial patterns of weather. There may not be any sense in which these differences “average out” between models.

Few studies have compared ecosystem impacts using more than one climate model. In a study of animal habitat suitability in Spain, Real et al. (2010) found that the differences in predicted habitat between two climate models for an emissions scenario were greater than the differences between scenarios using the same climate model. Given the large number of climate models and their difficulties modeling regional climate (noted above), it is apparent that vastly different ecological impacts could be predicted depending on the model used and the geographic region of interest, thereby violating criteria of rigor and reproducibility. One way around the problem is to use multiple models to sample the range of possible projections. This is especially important because at the regional scale some models may project wetter and some drier conditions. While this solution requires more work to perform an analysis, a valid result depends on some estimate of uncertainty.

### Selecting an ecosystem model and assessing uncertainty

Just as with climate models, ecosystem models can differ in their skill and bias as well as in their suitability for a particular task, and may produce widely varying results between models (Cramer et al. 2001). The reasons for choosing a particular model for an assessment need to be clearly stated. Process models can have considerable parametric uncertainty (e.g., Larocque et al. 2011) that can carry through the analysis to reduce outcome certainty. Ignoring these issues can make it look like model output is without error, whereas it is standard practice to report confidence intervals on experimental results.

Niche (suitability) models likewise are not guaranteed to be accurate (Segurado and Araújo 2004; Araújo and Guisan 2006). Classification accuracy of niche models does not tell the full story. Beale et al. (2008) and Pearson and Dawson (2003) provide a more complete discussion of the inherent uncertainty in these models including issues of variable covariance and spurious correlation. There is unfortunately no modeling method that has unambiguously been shown to be universally superior. It is critical to evaluate uncertainty due to these issues.

### Properly considering transient versus equilibrium responses

A large part of the ecosystem response literature consists of the analysis of habitat suitability (or geographic range) maps. Data on climate and other variables are used to predict presence of a species, forest type, or ecosystem using regression analysis or other tools. This model is then applied to future-modeled climates. A large shift in the range is usually considered to be an indicator of extinction risk because species, especially plants, are not able to migrate rapidly (e.g., Aitken et al. 2008; Morin et al. 2008).

Implicit in these conclusions is the equating of “suitable habitat” with the habitat necessary for survival (e.g., Araújo and Guisan 2006). However, the presence of a species means that it is competitive in the area where it occurs, not just that it can survive there. Small differences in competitive ability will eventually work themselves out and show up in distribution data, but this process may take a very long time, especially for long-lived organisms such as trees. Conditions outside the realized niche (i.e., where the species currently occurs) cannot be assumed to be lethal without proof. Projected climate changes are small compared to even daily temperature variation and, for most species, are not near lethal limits (e.g., Wertin et al. 2010). This general point was discussed in Loehle and LeBlanc (1996) but needs repeating because the confusion between transient and equilibrium response continues.

Multiple studies suggest that actual vegetation responses to even large climate shifts are slow. Cole (1985, 2009) documented a several thousand year transition in the Grand Canyon following past warming episodes. Cwynar and Spear (1991) showed that boreal forest advance in response to past warming took up to 1000 years, with a faster retreat due to cooling (which damages trees and prevents regeneration), as also shown by Tinner et al. (2007) for boreal dieback during the Little Ice Age. Williams et al. (2011) documented a slow late Quaternary boreal forest expansion across the Northern Hemisphere. In the boreal forest, where 20th century warming would suggest rapid geographic displacement, the response has been very slow (Masek 2001; Payette 2007). In no case has any study documented a “dead zone” in response to a past climate shift, as is implied by static habitat suitability models. Simulation models also suggest that such transitions should be gradual (Noble 1993; Loehle 2000, 2003).

Application of these criteria involves the careful delineation of implications from a study. It is not necessary that dynamic simulations be used. Rather, it is critical that information on transient response (relaxation times) can be used to qualify conclusions. If the study concerns mobile species, equilibrium might be quickly achieved, but if it concerns trees it could take hundreds to thousands of years. Nonoverlap of current and hypothetical geographic ranges does not mean extinction unless climate lethality can be proven by other means.

### Including effects of CO_2_ on plant response

CO_2_ enrichment directly increases growth and water-use efficiency (WUE) in plants (Medlyn et al. 2001; Leuzinger and Körner 2007; Loehle 2007; Kohler et al. 2010), with effects differing by taxa and CO_2_ level. Increased WUE is particularly important when water is limiting, and has been shown to mitigate considerable drought stress (e.g., de Graff et al. 2006; Wertin et al. 2010). WUE is not considered here in terms of hydrology but only with respect to drought response. Free-air CO_2_ exchange (FACE) and open top chambers have both shown a positive response of plant growth to CO_2_ enrichment (Curtis and Wang 1998; de Graaf et al. 2006). For plant growth or habitat suitability models, failure to include CO_2_ effects and WUE increases with time over the next 100 years will lead to significant overestimates of negative impacts of elevated temperature, reduced moisture, or both together (Cramer et al. 2001; Pan et al. 2009; Friend 2010). Mechanistic models that incorporate CO_2_ effects are generally based on experimental results such as FACE experiments, in some cases with medium-sized trees over many years. Keenan et al. (2011) simulated forest growth with and without CO_2_ effects. Without including CO_2_ effects in the simulation, all three species showed decreased net primary productivity (NPP) over time under the warming scenario. However, when CO_2_ effects were included, the species exhibited increased growth until about 2070 followed by a slight decline, ending at 2100 with slightly higher NPP than in 2000. How CO_2_ effects are addressed can make the difference between positive and negative growth responses under many scenarios. It is also the case, of course, that no CO_2_ experiments have gone as far as 100 years, covered large spatial extents, or allowed for species shifts, so models incorporating CO_2_ effects may give unrealistic or exaggerated responses at some time/space scales (Leuzinger et al. 2011).

### Evaluating implications of simplifying assumptions

In order to conduct a geographic analysis of climate effects, it is often necessary to make simplifying assumptions due to data resolution. We may, for example, have GCM output at 1°× 1° resolution. In the real world, however, fine-scale topographic effects moderate regional climate in complex terrain. An analysis based on coarse-scale data might suggest that no suitable habitat remains under some scenario, when in fact topography might provide for refugia (Scherrer and Körner 2010; Dobrowski 2011; Godfree et al. 2011). Other simplifications might be made, such as using a single very dry soil type for the simulation (e.g., Coops and Waring 2011). The criterion here is that such simplifications, while perhaps necessary because of data or computational limitations, have implications that qualify the results and should be discussed. In addition, geographic data can have errors resulting from vegetation classification, measurement error and bias, aggregation error, and other sources, leading to effects possibly larger than those resulting from the scenarios being evaluated (Verburg et al. 2011).

### Considering animal linkage with vegetation

When suitability/range map-type models are developed for plants, there are good reasons to suppose that climate directly determines plant distribution (as modified by competition and fire, or course). It is equally easy to develop models for animals’ range, but these models might actually be proxies for the vegetation on which the animals depend as much as for climate per se. That is, spurious correlation is a real danger in the absence of experimental data. For example, ruminants depend on grasslands and waterfowl depend on marshes and lakes. If an animal depends on certain vegetation that exhibits a long lag in responding to climate, the animal may likewise persist in its current range unless its physiological tolerance is actually exceeded. If the niche model uses climate variables, the projection of animal response could be unrealistic. Thus, it should be recognized that suitability models are correlational rather than fundamental, and investigators should carefully consider model output in this light.

## Literature Survey

A literature survey was conducted to evaluate compliance with the assessment criteria suggested here. All issues of*Global Change Biology*in 2009 and 2010 and the first four issues in 2011 were assessed. All terrestrial studies discussing future impacts on species or ecosystems were considered (not carbon or fire studies). Experimental and field studies were outside the scope of the review. A total of 20 papers were evaluated (Table 1), with one study evaluated under two categories. In the author's experience, these 20 papers are similar to others in the field and serve to illustrate the points made.

**Table 1:** Percent compliance with criteria for literature survey cases (percentages calculated only for criteria relevant to the studies in question). NA reflects that criterion not relevant to that model type

	Criteria^a^									
	
	*n*	1	2	3	4	5	6	7	8	9
Dynamic models										
Plant	7	1.0	0.86	0	0.57	0.14	1.0	0.71	1.0	NA
Animal	2	0	0.5	0	0.5	1.0	1.0	NA	0	0
Niche models										
Plant	7	0.71	0.43	0	0.43	0.43	0	0	0.57	NA
Animal	5	0.8	0.6	0	0.4	0.4	0	0	0	0.6

NA = not applicable

a criteria areas follows:

(1) Clearly define emissions scenario.

(2) Select climate model(s).

(3) Evaluate climate model skill and bias.

(4) Quantify GCM between-model variability.

(5) Select an ecosystem model and assess uncertainty.

(6) Properly consider transient versus equilibrium responses.

(7) Include effects of CO_2_ on plant response.

(8) Evaluate implications of simplifying assumptions.

(9) Consider animal linkage with vegetation.

### Growth models

Nine papers (seven of vegetation) with some sort of dynamic or growth model utilized a variety of approaches and often met most of the criteria proposed for impact assessments. Most used the most recent IPCC AR4 climate simulations. Keenan et al. (2011) did underestimate outcome variability by only using one climate simulation and one forest growth model, but is the only study that directly compared niche and growth model results (using multiple niche models but a single vegetation model). The inclusion of CO_2_ effects led to an opposite conclusion on impacts (positive) compared to leaving them out (negative). Doherty et al. (2010) evaluated effects of future climate on NPP of East Africa. They used output from nine GCM models to capture uncertainty but only a single vegetation model that did simulate CO_2_ enrichment effects. All cases projected increased NPP in the study area. Poulter et al. (2010) simulated response of the Amazon forest to CO_2_ and eight different GCMs as well as vegetation model parameter uncertainty. They found that between-climate-model differences dominated outcome spread compared to CO_2_ or vegetation model parameter uncertainty, with future precipitation uncertainty the greatest unknown determinant of outcomes.

Chen et al. (2010) evaluated response of Douglas-fir to projected climate change using a tree ring index model of growth response. Variability was captured by using output from five climate models. The inability of this approach to include effects of rising CO_2_ is problematic because the main impacts of climate change in their analysis were manifested via drought stress, which rising CO_2_ and thus increasing WUE would help ameliorate. Xu et al. (2009) simulated forest growth in northern Minnesota using a single process model. CO_2_ was included in the model. Future climate data (27 profiles) were mostly from older GCMs used in the third IPCC assessment in 2001. The large number of climate runs allowed the large uncertainty due to forecasting to be quantified. Morin et al. (2009) used a process-based statistical model for leaf unfolding for 22 North American tree species as a function of climate. A single GCM run from 2001 was used for the A2 and B2 scenarios only. The effect of CO_2_ on phenology was not assessed. Spatial resolution was probably not an issue. With a single older GCM run, there is no way to evaluate the representativeness of their forecasts. Scheiter and Higgens (2009) used a mechanistic model of grassland and forest to evaluate response of the vegetation of Africa to climate change. The model included CO_2_ effects that were believed to account for increased forest area and biomass as well as the increase in total vegetated land (at the expense of desert) by 2100. Spatial resolution was good at the scale of 1 ha. The GCM used was a 2007 model run, but the lack of intermodel comparison makes it difficult to evaluate results.

Two animal process models were found. Gilg et al. (2009) explored effects of future climate on Arctic predator–prey systems. The climate scenarios were qualitative and hence difficult to evaluate. Snäll et al. (2009) used seven up-to-date GCM models and a process model for plague in prairie dogs. By sampling from the plague model parameter uncertainty space as well as the multiple GCMs they were able to do an exemplary job quantifying uncertainty, although they did not assess GCM bias or skill.

Overall, process-based studies did a good job assessing uncertainty due to between climate model variation and within ecosystem model parametric uncertainty, although only Snäll et al. (2009) and Poulter et al. (2010) evaluated both. Most used current climate models but none evaluated GCM skill and bias issues.

### Niche models

All seven studies using niche models for plant distributions (Bradley 2009; Bradley et al. 2009; Randin et al. 2009; Feeley and Silman 2010; Dirnböck and Rabitsch 2011; Dlamini 2011; Keenan et al. 2011) failed to consider CO_2_ effects on future growth. Because much of the impact of future climate on plants results from net water deficiency, the lack of consideration of CO_2_ effects calls results into question, especially in light of Keenan et al.'s (2011) results discussed above. Use of future climate scenarios was mixed. Keenan et al. (2011) used IPCC AR4 climate model results, but Dlamini (2011) used a result from 2000, which is rather old. Dirnböck and Rabitsch (2011) and Feeley and Silman (2010) used simple increases in temperature, including an extreme 8°C rise in the latter case, with no increase in precipitation. Failure to consider future precipitation changes generally will make impacts more negative for plants. Uncertainty due to niche model differences was considered by Keenan et al. (2011), and that due to between-climate model differences by Dlamini (2011). The equilibrium assumption (equating niche model changes with immediate population changes) was universal in these studies, with only a mention of possible lags by Dirnböck and Rabitsch (2011), although possible lags were considered to be in terms of mere decades. Two studies of invasive plants (Bradley 2009; Bradley et al. 2009) using niche models used 10 AR4 GCM runs and were thus able to quantify model-based uncertainty, but did not assess model skill or bias and did not clearly cite the model runs used. Because they only evaluated potential habitat for invasion, there was less problem with the equilibrium assumption, but they did assume that future unsuitable habitat for the invasive species would immediately become a conservation opportunity. Randin et al. (2009) compared scenarios at 50 km × 50 km versus 25 m × 25 m plot scales. Because they interpolated climate to account for topography at the smaller plot size, the local-scale models showed persistence of up to 100% of species that the European-scale model predicted would lose their entire habitat. The study was limited by the use of (apparently) an older single (approximately 2000) GCM run. The net effect in all these models of excluding the beneficial effects of elevated CO_2_ and assuming rapid vegetation changes due to changes in available suitable habitat is to greatly amplify likely negative effects or even to convert positive effects into negative ones, as in Keenan et al. (2011). Likewise, niche models at too coarse a resolution will miss refugia from climate change.

Five niche models were used to assess animal distributions (Jarema et al. 2009; Carroll et al. 2010; Carvalho et al. 2010; Rebelo et al. 2010; Habel et al. 2011). These studies used climate projections from 2004 or 2005 except for Carvalho et al. (2010) for which the date of the climate model could not be determined and Jarema et al. (2009), which used a 2001 GCM run. Carvalho et al. (2010) used more than one niche model (nine) and GCM (three) and Jarema et al. (2009) used two GCM models and multiple niche models. Carroll et al. (2010) developed animal niche models that included vegetation and held vegetation constant based on consideration of lags that were likely to occur in forests of the Pacific Northwest United States. Jarema et al. (2009) found niche models based on habitat to be almost as good as those using climate variables alone. All other studies used niche models dominated by climate variables and thus ignored the possibility that animals may be found in their current habitat based on the vegetation, which would probably respond to climate change in a lagged fashion. They all further assumed equilibrium response, whereas animal distributions are likely to be heavily influenced by competition and predation effects that would take time to come to equilibrium in a new climate. Finally, a fairly extreme scenario of 6°C warming was used by Rebelo et al. (2010). Simplifying assumptions that may have affected results include use of 35 km^2^ grid cells in Dirnböck and Rabitsch (2011), only a simple temperature gradient (no heterogeneity or precipitation) in Feeley and Silman (2010), the coarse spatial scale used in Carvalho et al. (2010) that would not allow for refugia, and the assumption in Rebelo et al. (2010) that bats are dispersal-limited. Overall, uncertainty was underestimated and effects were probably exaggerated in this set of studies.

## Discussion

The criteria suggested in this study are intended to guide decisions in assessments of future climate change impacts and to help prevent results from being arbitrary or showing false precision due to lack of uncertainty information. The literature survey revealed that recent studies have not considered some of the proposed criteria (Table 1). Violation of any of the criteria can be serious, but some are more easily remedied than others. The criteria are next considered in turn, with suggestions for improved assessments.

The examined papers generally referred back to IPCC AR4 emissions scenarios (IPCC 2007). Usually, either the A2 scenario was used or a range of scenarios was evaluated. This standard set of scenarios helps with interstudy comparisons. Some studies used arbitrary temperature increases rather than model outputs, which has the added problem that precipitation was held constant or ignored. If simple temperature increases are evaluated, they should at least be more clearly related to model scenarios. It is difficult to justify leaving precipitation changes out of an assessment and not difficult to add this factor. Recent generation climate simulations were not necessarily used, although the growth model studies mostly used up-to-date results. Four of seven plant niche models used out-of-date climate models. There is really no good reason to use old climate simulations.

No study took into account the skill or bias of the climate models. This is problematic, especially because many used models for current ecosystems calibrated with actual weather data and compared them to future simulated climates in which a bias (offset) could exist. At least a qualitative assessment of climate model outputs compared to the study area seems necessary before doing an assessment. Of the sources of uncertainty (climate model, and niche model or growth model), only three studies (Jarema et al. 2009; Carvalho et al. 2010; Poulter et al. 2010) considered both and many studies included only a single model and GCM dataset. It is not impossible to avoid this problem by explicitly using the multiple climate model outputs, which are becoming increasingly available for regional scales. Likewise, ensemble means of climate data might be available and ensemble means or overlap for niche models (Araújo and New 2007) are not difficult to develop, since the statistical tools are widely available.

Studies using niche models mostly failed to properly consider lags in vegetation response to climate change. They assumed that habitat defined as “unsuitable” by the niche model was immediately uninhabitable by the species or vegetation without presenting evidence that the altered climate would be actually lethal. Instead, competitive displacement, an inherently slow process that may take centuries (Noble 1993; Loehle 2000, 2003), could eventually result in the changes suggested by niche models. While niche models are inherently equilibrium based, it is possible to perform some assessment of nonequilibrium response (e.g., Araújo and Pearson 2005). In addition, data do exist on drought and heat tolerance of many species, and such data should be consulted before making claims that species or ecosystems will perish/vanish over the next few decades due to a few degrees warming. If the temperature rise is within the thermal tolerance of a species, then range shifts, not extinctions, are going to result and may take centuries. Consulting the literature to evaluate the likely time dynamics of the output of niche models is not a burdensome requirement.

Most studies using growth models included beneficial effects of elevated CO_2_ on plant growth. It is noteworthy that these models forecast much less impact or even positive effects compared to niche models, which are inherently unable to account for future elevated CO_2_ effects. While the exact effects of elevated CO_2_ over the long term are not known (e.g., Leuzinger et al. 2011), the experimental literature seems to support a growth-enhancement conclusion and models have widely adopted this result. It would seem that dynamic (mechanistic) models of plant response to climate change should be preferred, though niche models are much more widely published, perhaps due to simplicity and cost. Cost alone does not justify a method that leads to the wrong answer. At the very least, the result of any niche analysis needs to be qualified by discussion of how CO_2_ effects would alter the result.

The primary simplifying assumption that impacted results in a number of studies was the reduction of spatial heterogeneity considered, either due to grid scale or to elevational gradient representation (e.g., representing it as a smooth curve). Spatial heterogeneity can provide refugia, and failure to consider this effect will tend to exaggerate impacts, as clearly shown by Randin et al. (2009). The limiting factors for performing more spatially resolved analyses are computer time and input data. The former can be overcome by distributed computing or weekend runs. The latter is at least mitigated by using the highest resolution data available rather than aggregated data. The conceptual and analysis steps are identical in either case.

Finally, in animal impact studies, a pervasive trend was to model animal response solely as a function of climate. If animals select habitat based on vegetation and the response of vegetation to changing climate is lagged, the response of animals will probably also be lagged. The assumption that animals are limited by climate as defined by a niche model is virtually unverified.

When study results can be completely altered by including or excluding a factor such as CO_2_ or spatial heterogeneity and when between-model forecasts can be extremely variable, it is essential that these and other known factors be recognized and addressed in any analysis of climate change effects. The majority of surveyed studies might have yielded very different results if the criteria suggested here had been applied. More attention to criteria such as those proposed would lead to much more reliable and useful impact assessments. It has been shown above that most of these problems are easily fixed, require a modest effort (e.g., using niche model ensembles), or can be dealt with in the discussion of results.
